# Mechanisims of asthma and allergic disease – 1081. Resolvin inhibits the cryopyrin/nalp3 inflammasome

**DOI:** 10.1186/1939-4551-6-S1-P77

**Published:** 2013-04-23

**Authors:** Cox Ruan, Narasaiah Kolliputi

**Affiliations:** 1 Internal Medicine, University of South Florida, Tampa, USA

## Background

The inflammasome is a novel protein complex that stimulates caspase-1 activation to promote the processing and secretion of IL-1β, a pro-inflammatory cytokine, which is among the most biologically important inflammatory mediators in allergic airway diseases. Among the various types of inflammasomes, Cryopyrin/NALP3 has been suggested to be involved in sensing sterile stress response, extracellular ATP, danger-associated molecular patterns (DAMPs), and crystals. Recently inappropriate NALP3 inflammasome activity has been reported in various allergic airway diseases including asthma. Therefore, inhibitors of the NALP3 inflammasome offer considerable therapeutic promise. Recently Omega-3 fatty acid derivates termed resolvins have shown to alter the effects of pro-inflammatory cytokine storm seen in inflammatory diseases. However the ability of resolvins to modulate the effects of inflammasome activation has not been studied. We investigated whether resolvin treatment inhibits inflammasome activation and regulates the functional effects of inflammasome mediated by IL-1β secretion.

## Methods

In our study the inflammasome was activated by ATP and H_2_O_2_ (known nflammasome activators in acute lung injury) in the absence or presence of D series resolvins, resolvin D1 and resolvin D2. Inflammasome activation was assessed by analyzing IL-1β release (end product of inflammasome activation) and caspase-1 cleavage (indicator of inflammasome activation) in THP-1 cells. Further inflammasome was activated in the presence or absence of resolvin in THP-1 cells, and supernatants from these cells were added to A549 cells and human primary small airway epithelial cells (HPSAEC) to study inflammasome mediated functional effects.

## Results

Our results indicate that resolvin treatment ameliorates inflammasome activation as indicated by decreased caspase-1 activity and IL-1β release. In addition resolvin treatment inhibits inflammasome mediated epithelial cell activation as indicated by suppressed IL-8 release and decreased and ICAM expression.

## Conclusions

These novel findings suggest that resolvins can be used to modulate the inflammasome activity as well as blunt the effects of the IL-1β mediated cytokine storm stemming from inflammasome activation. These results may offer a therapeutic approach to airway diseases such as asthma, where ungoverned pro-inflammatory cytokine secretion exacerbates the disease pathology.

